# Perioperative chemotherapy more of a benefit for overall survival than adjuvant chemotherapy for operable gastric cancer: an updated Meta-analysis

**DOI:** 10.1038/srep12850

**Published:** 2015-08-05

**Authors:** Ya’nan Yang, Xue Yin, Lei Sheng, Shan Xu, Lingling Dong, Lian Liu

**Affiliations:** 1 Department of Chemotherapy, Cancer Center, Qilu Hospital, Shandong University, Jinan, China; 2 Cancer Therapeutics Laboratory, Centre for Personalized Cancer Medicine, School of Medicine, University of Adelaide, Australia; 3 Department of Cancer, Weifang Traditional Chinese Medical Hospital, Weifang, China

## Abstract

To clarify the effect of neoadjuvant chemotherapy (NAC) on the survival outcomes of operable gastric cancers, we searched PubMed, Embase, and Cochrane Library for randomized clinical trials published until June 2014 that compared NAC-containing strategies with NAC-free strategies in patients with adenocarcinoma of the stomach or the esophagogastric junction, who had undergone potentially curative resection. The adjusted pooled hazard ratio (HR) for overall survival (OS) was insignificant when comparing the NAC-containing arm with the NAC-free arm. Subgroup analysis showed that the OS of the treatment arm that involved both adjuvant chemotherapy (AC) and NAC was significantly improved over the control arm (AC only) (HR = 0.48, 95% CI: 0.35–0.67; *P* < 0.001). While NAC alone plus surgery did not show any survival benefit over surgery alone. Perioperative chemotherapy (PC) also showed a significant increase in PFS and a significant reduction in distant metastasis compared to surgery alone. Therefore, in patients with resectable gastric cancer, NAC alone is not enough and AC alone is not good enough to definitely improve their OS. Collectively, PC combined with surgery could maximize the survival benefit for patients with resectable gastric cancer.

Although there has been a decline in the incidence and mortality of gastric cancer in the past few years, this form of cancer remains a common and very fatal disease and is still the second leading cause of cancer-related deaths worldwide^1^. The general prognosis for gastric cancer is poor, and the 5-year survival rate is less than 20%^2^. This might result not only from the difficulty in making an early diagnosis but also from the high rate of recurrence and metastasis. At present, surgery is still the only curative treatment for gastric cancer. However, relapse and metastasis still tend to occur in 40%–60% of cases, even after an R0 gastrectomy^3^,^4^. To overcome the limitations of surgery in improving the prognosis of gastric cancer, perioperative therapies, which include pre- or postoperative chemotherapy or radiotherapy, have been studied and given much attention in recent years. Based on several multicenter, randomized controlled trials (RCTs), different perioperative treatments have been used in different geographic regions. According to the results of the Adjuvant Chemotherapy Trial ACTS-GC^5^,^6^ and CLASSIC^7^, adjuvant chemotherapy (AC) has been widely used following D2 gastrectomy in East Asia, where there is a high incidence of gastric cancer. In the US, chemoradiotherapy has been recommended to treat locally advanced gastric cancer based on the US Intergroup-0116 trial^4^. In parts of Europe, perioperative chemotherapy (PC) is regarded as the standard treatment on the basis of the MAGIC trial^3^.

Although different perioperative therapies are used in different regions according to relevant RCTs with regional differences, the effects on improving the survival of gastric cancer patients remain limited. Neoadjuvant chemotherapy (NAC) was once considered a strategy that might have survival benefits in patients with locally advanced gastric cancer. However, in contrast to AC, which has been widely recommended, NAC has been found to be successful only in combination with AC (i.e. PC) in Europe^3^,^8^. Thus, the role of NAC in gastric cancer is still controversial. To date, most clinical trials or meta-analyses have shown that NAC can improve the R0 resection rate in patients with locally advanced gastric cancer, but the results regarding long-term efficacy such as overall survival (OS) and progression-free survival (PFS) have been inconsistent^9^,^10^,^11^,^12^. Furthermore, there were some defects in these meta-analyses, such as using odds ratio (OR) instead of hazard ratio (HR) to estimate long-term survival effect, combining trials with great heterogeneity, including unqualified trials, lack of quality evaluation, and inclusion of non-random controlled trials, all of which made the conclusions less reliable.

Would NAC alone have survival benefits for operable gastric cancer patients, or should it be combined with AC? Or is AC itself sufficient to improve the survival in gastric cancer patients and NAC is not useful? How do we look upon the use of NAC in Asia where the highest incidence of gastric cancer, skillful operative technique, and good adjuvant treatment strategies coexist? The key question is that we still don’t know if PC exactly has an extra advantage than AC in the treatment of operable gastric cancers. To address these questions, we performed an updated meta-analysis involving 2,093 patients from 14 different trials between 1966 and June, 2014, comparing NAC-containing strategies with NAC-free strategies, mainly in terms of OS of patients with resectable gastric cancer, estimated by HR and 95% CI.

## Methods

### Literature Search

We searched PubMed, Embase, ASCO and ESMO meeting abstracts, and Cochrane Library by computer for randomized clinical trials published until June 2014 that compared NAC-containing strategies with NAC-free strategies in patients with histologically proven adenocarcinoma of the stomach or the esophagogastric junction, who had undergone potentially curative resection. The search used the following key words: stomach cancer, gastric cancer, stomach tumor, gastric tumor, stomach neoplasm, gastric neoplasm, neoadjuvant chemotherapy, preoperative chemotherapy, primary chemotherapy, neoadjuvant treatment, neoadjuvant therapy, primary treatment, preoperative treatment, primary therapy, and preoperative therapy. The search term for MEDLINE was as follows: (resect* OR operative) AND ((treatment OR therapy OR chemotherapy) AND (neoadjuvant OR preoperat* OR perioperat*) AND (tumor OR tumour OR neoplas* OR cancer OR carcinoma) AND (gastric OR stomach OR esophag*)). Relevant articles and abstracts were reviewed, and the reference lists from these sources were searched for additional trials. Where results were reported or updated in more than one publication, only the most recent publication was used. There was no language restriction.

### Data extraction

Two authors extracted the outcome data independently using a pre-designed data extraction form. Authors, year of publication, country of patients, sample size (per arm), chemotherapy regimen, surgical procedure, follow-up period, treatment effect (OS, PFS, survival rate, rate of macroscopic radical resection, tumor stage at pathological examination, local regional recurrence, and distant metastasis), and adverse events (postoperative morbidity and mortality) of each eligible trial were recorded. The last name of the author was used to represent the corresponding study. If more than two authors had the same last name, the initial of the first name was added to make a distinction.

### Statistical analysis

The primary endpoints of this meta-analysis were OS (the time from random assignment to the last follow-up or death) and PFS (the time from random assignment to objective tumor progression or death) or disease-free survival (DFS: the time from random assignment to tumor recurrence or death). Secondary endpoints were R0 (margin negative) resection rate or curative resection rate, down-staging effect, incidence of recurrence, 5-year survival rate, and postoperative morbidity and mortality.

Results regarding OS and PFS were expressed as HR with 95% CIs, which were used directly or estimated from the Kaplan-Meier survival curves or by the indirect method described by Parmer and colleagues^13^. The results for the 5-year survival rate, R0 resection rate, postoperative morbidity and mortality, incidence of recurrence, and metastasis, were expressed as relative risk (RR) and CIs. Moreover, to assess the down-staging effect of NAC (that is, the probability of having a less advanced stage of the disease at pathological examination at the time of surgery), we separated patients with negative nodes from those with positive nodes on pathological examination after surgery. Also, the percentage of patients in primary tumor stage T0-2 was calculated. All analyses were carried out based on the intent-to-treat principle except where only evaluable patients’ data were available.

χ^2^ tests were performed to study heterogeneity between trials, and the *I*^2^ statistic was used to estimate the percentage of total variation across studies. Heterogeneity was considered for an *I*^2^ statistic greater than 50% or a *P*-value for the Q-test of less than 0.1. In the present meta-analysis, the results using the random-effect model are presented to take into account the possible clinical diversity and methodological variation among the studies. The number needed to treat (NNT) was applied for outcomes with a statistical difference. Publication bias was checked by visual inspection of contour-enhanced funnel plots^14^ and Egger’s test^15^ with the slope of the regression (bias coefficient) indicating the extent of any bias. All *P*-values were two-sided and *P* < 0.05 was considered significant. All statistical analyses were performed with the Stata statistical software package (release 12·0; Stata Corporation, College Station, TX, USA).

## Results

### Study selection and characteristics of included studies

**Figure 1:**
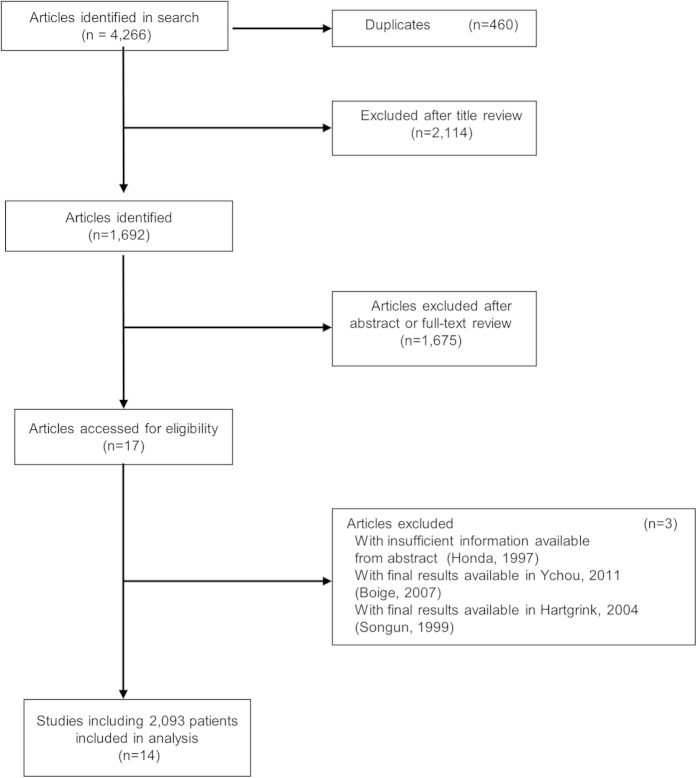
Flow diagram showing inclusion and exclusion of studies.

Figure 1 shows the flow of the trial selection process. In brief, 4,266 studies were identified from the searches and 14 trials were included in the meta-analysis after the abstracts and full texts had been screened finally. The 2,093 gastric cancer patients who had been enrolled were placed in the NAC group (n = 952) or the control group (n = 1,141). Table 1 shows the characteristics of the 14 trials included in the analysis. We divided them into four categories based on the therapeutic strategy: (1) NAC plus surgery in the treatment arm compared with surgery only in the control arm^16^,^17^,^18^,^19^,^20^; (2) PC plus surgery in the treatment arm compared with AC following surgery in the control arm^21^,^22^,^23^,^24^,^25^; (3) PC plus surgery in the treatment arm compared with surgery only in the control arm^3^,^26^,^27^ and (4) NAC plus surgery in the treatment arm compared with AC following surgery in the control arm^28^. It should be noted that the term “NAC arm” is used hereafter to stand for interventions containing NAC in the treatment arm, and this refers to both category 1 and 2 above.

### Quality assessment of included trials

The methodological quality of each study that was included was assessed according to the Cochrane Handbook for Systematic Review of Interventions 5.1.0.^29^ for the following six requirements: the method of randomization, allocation concealment, blindness, selective reporting, baseline, and completeness of follow-up. Five studies were graded A (low risk of bias), eight studies were graded B (moderate risk of bias), and one study was graded C (high risk of bias) (Table 2).

### Overall survival

**Figure 2:**
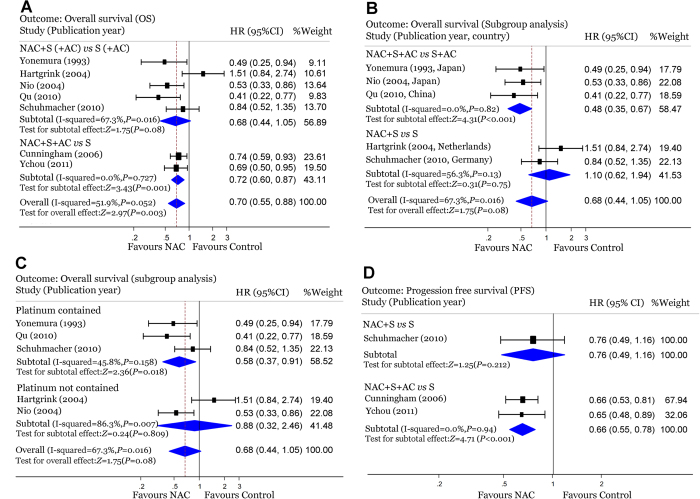
Overall HR for survival outcomes of resectable gastric cancers when comparing NAC-containing arm and NAC-free arm. (**A**) Forest plot showing the OS of resectable gastric cancer patients in the seven NAC-containing RCTs. (**B**) Forest plot showing the OS of resectable gastric cancer patients in the five NAC-containing RCTs subgrouped by therapeutic strategies. (**C**) Forest plot showing the OS of resectable gastric cancer patients in the five NAC-containing RCTs subgrouped by whether or not the regimen contained platinum. (**D**) Forest plot showing the PFS of resectable gastric cancer patients in three NAC-containing RCTs. HR, hazard ratio; 95% CI, 95%confidence interval; OS, overall survival; PFS, progression-free survival; NAC, neoadjuvant chemotherapy; AC, adjuvant chemotherapy; S, surgery; RCTs, randomized controlled trials.

The HR and 95% CIs of OS could be obtained directly^3^,^20^,^24^,^25^,^27^ or indirectly^19^,^22^ in seven of the eight full-text articles investigating NAC in operable gastric cancer patients. The adjusted pooled estimate of the treatment effect appeared to be statistically significant (adjusted HR for death, 0.70; 95% CI: 0.55–0.88; *P* = 0.003; Fig. 2A). However, the therapeutic strategies in the seven trials varied geographically to some extent. Only two trials^19^,^20^ focused primarily on NAC alone with the treatment arm receiving NAC plus surgery and the control arm receiving surgery alone. Trials by Nio^24^, Yonemura^22^, and Qu^25^ had PC (i.e. NAC and AC) plus surgery in the treatment arm and AC following surgery in the control arm. Because the only variation was whether or not NAC was performed, these three trials were regarded as being qualified in evaluating the effect of NAC and had been combined with the two trials above. However, the rest two trials^3^,^27^ compared the effects of PC plus surgery and surgery alone. It was impossible to determine the relative contribution of NAC regarding survival benefit. As shown in Fig. 2A, the pooled HR for OS in these two trials was 0.72 (95% CI: 0.60–0.87; P = 0.001), revealing a substantial survival benefit. Considering the high weight of these two trials in this meta-analysis (43%), the evaluation of the effect of NAC was interfered with by the performance of AC. Therefore, we excluded these two trials and combined the other five trials. The pooled, adjusted estimate of the treatment effect then became insignificant (HR = 0.68; 95% CI: 0.44–1.05; *P* = 0.08; Fig. 2B), although there was a trend toward favoring NAC. In addition, exclusion of the study^24^ with high risk of bias doesn’t have significant impact on the pooled estimate of the treatment effect (HR = 0.74; 95% CI: 0.41–1.25; *P* = 0.248).

Then we performed a subgroup analysis of these five trials based on whether or not AC had been given. The pooled HR for the three trials^22^,^24^,^25^ that involved AC in both the treatment arm and the control arm was 0.48 (95% CI: 0.35–0.67; *P* < 0.001; Fig. 2B) indicating that the OS of the treatment arm (PC and surgery) had a significant benefit than that of the control arm (AC and surgery). When the study^24^ having high risk of bias was omitted, the pool HR for the rest of two trials^22^,^25^ remained significant (HR = 0.45; 95% CI: 0.28–0.70; *P* = 0.001). In the other two trials without AC in the treatment and control arms, NAC plus surgery did not give any survival benefit over surgery alone (Fig. 2B). By coincidence, the three trials comparing PC with AC were all conducted in Asian countries (China and Japan) while the other two comparing NAC with surgery were both conducted in European countries (the Netherlands and Germany). Another subgroup analysis was performed to evaluate the different regimens used in NAC. The platinum-containing regimens showed better efficacy in improving OS than other regimens (Fig. 2C).

### Progression-free survival

For PFS, data could be extracted from the descriptions of three RCTs, including the two comparing PC with surgery and an EORTC40954 trial comparing NAC with surgery alone. The results of the perioperative subgroup showed a significant increase in PFS^3^,^27^. The HR of 0.66 (95% CI: 0.55–0.78; *P* < 0.001) represented a 34% overall relative reduction in the risk of disease progression from PC. There appeared to be no potential survival benefit in the EORTC40954 trial, which only performed NAC in the treatment arm (Fig. 2D)^20^.

### R0 resection rate

**Figure 3:**
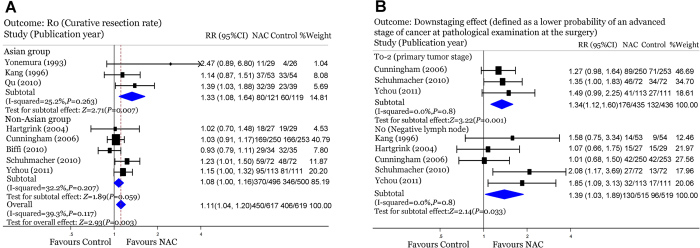
Pooled analysis of local control effect of NAC for gastric cancers. (**A**) Forest plot showing the R0 resection rate of gastric cancer patients in eight NAC-containing RCTs. The relative risk (RR) with 95% CI for effect of treatment on R0 rate is shown on a logarithmic scale using a fixed effect model. Subgroup analysis was based on geographic regions. (**B**) Forest plot showing the down-staging effect of gastric cancer patients in eight NAC-containing RCTs. The RR with 95% CI for effect of treatment on down-staging is shown on a logarithmic scale using a random effect model.

The R0 resection rate for gastric cancer was reported in eight trials. It was higher for the NAC group than for the control group (72.9% *vs*. 65.6%) with an RR of 1.11 (95% CI: 1.04–1.20; *P* = 0.003; Fig. 3A). Subgroup analysis revealed that in the Asian group the R0 resection rate in the NAC arm was higher than that in the control arm (66.1% *vs*. 50.4%) and reached statistical significance with a pooled RR of 1.33 (95% CI: 1.08–1.64; *P* *=* 0.007; NNT = 7; Fig. 3A)^21^,^22^,^25^. In non-Asian groups, the R0 resection rate was 74.6% in the NAC arm and 69.2% in the control arm^3^,^19^,^20^,^27^,^28^. Although not reaching statistical significance (RR = 1.08, 95% CI: 1.00–1.16; *P* = 0.059; NNT = 19; Fig. 3A), there was still a trend indicating the effectiveness of NAC in improving R0 resection rate.

### Down-staging effect

A total of eight RCTs involving 1,905 patients were available for evaluation of the down-staging effect of NAC (Fig. 3B). The percentage of patients achieving negative lymph node status was 25.2% in the NAC group and 18.5% in the control group (RR = 1.39; 95% CI: 1.03–1.89; NNT = 15; *P* = 0.033)^3^,^19^,^20^,^21^,^27^. Similar results were obtained when a fixed-effect model was used (RR = 1.37; 95% CI: 1.09–1.73; *P* = 0.007). Furthermore, the rate of T0-2 was higher for the NAC group than for the control group (40.5% *vs.* 30.3%), with an RR of 1.34 (95% CI: 1.12–1.60; *P* = 0.001) and an NNT of 10^3^,^20^,^27^.

### Five-year Survival Rate

Four studies compared the 5-year survival rate in the two arms. The pooled data showed no significant increase in 5-year survival rate of 52.8% (104/197) in the NAC arm and 48.7% (129/265) in the control arm (RR = 1.08, 95% CI: 0.90–1.28; *P* = 0.406). Among these, three trials used NAC plus surgery in the treatment arm and surgery alone in the control arm and one trial performed PC plus surgery in the treatment arm and AC following surgery in the control arm, so separate analyses were conducted. The results showed that neither the three AC-free trials (RR = 1.18; 95% CI: 0.90–1.56; *P* = 0.221) nor the AC-containing trial (RR = 0.98, 95% CI: 0.78–1.23; *P* = 0.863) showed any statistical significance, and this indicated that NAC alone might not suffice to improve the 5-year survival rate of resectable gastric cancer (Supplemental Fig. 1).

### Postoperative Morbidity and Mortality

From five trials, the incidence of overall postoperative morbidity was reported as 34.0% (172/506) in the NAC group and 30.8% (157/510) in the surgery alone group, but this difference between the two arms was not significant (RR = 1.11, 95% CI: 0.93–1.31; *P* = 0.24). Similar results were obtained when a random effect model was used (RR = 1.08, 95% CI: 0.91–1.28; *P* = 0.38). The incidence rate of postoperative mortality reported from six studies was 5.0% (26/525) in the NAC group and 4.4% (23/527) in the surgery group. Combined data showed no significant difference in postoperative mortality between the two arms (RR = 1.13, 95% CI: 0.66–1.93; *P* = 0.66). Similar results were obtained when a random effect model was used (RR = 1.09, 95% CI = 0.63–1.90; *P* = 0.75) (Supplemental Fig. 2).

### Recurrence and metastasis

**Figure 4:**
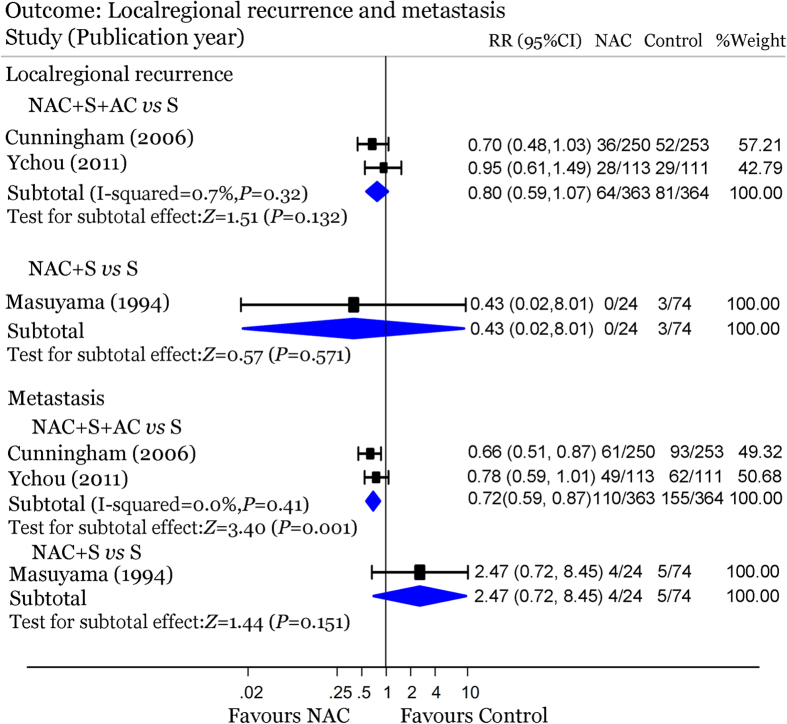
Forest plot showing loco-regional recurrence and metastasis of gastric cancer patients in three NAC-containing RCTs.

As showed in Fig. 4, the rate of distant metastasis was 30.3% in the PC arm and 42.6% in the surgery arm, with an RR of 0.72 (95% CI: 0.59–0.87; *P* = 0.001), showing a significant reduction in distant metastasis associated with PC over surgery. Significant advantage in loco-regional recurrence of PC arm was not reached (RR = 0.80; 95% CI: 0.59–1.07; *P* = 0.132). In the trial comparing NAC with surgery alone^16^, neither the loco-regional recurrence rate (RR = 0.43, 95% CI: 0.02–8.01; *P* = 0.571) nor the distal metastasis rate (RR = 2.47, 95% CI: 0.72–8.45; *P* = 0.151) showed any statistical significance.

### Publication Bias

**Figure 5:**
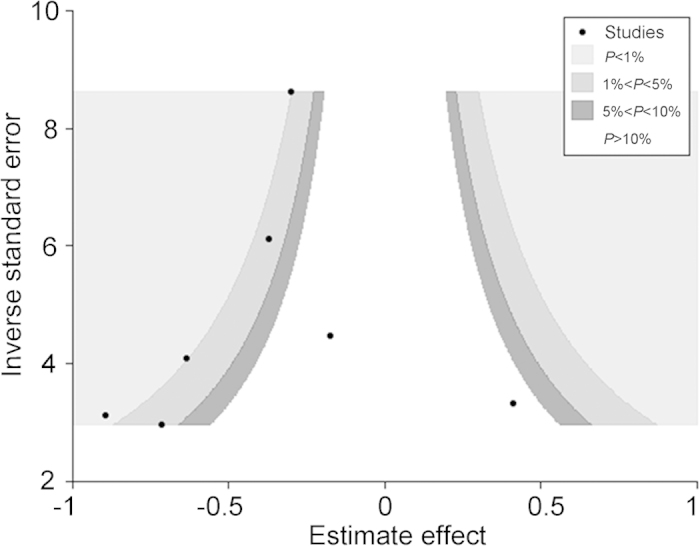
Contour-enhanced funnel plot for meta-analysis of RCTs of NAC for resectable gastric cancers. The “missing” studies would be expected to lie in areas of high statistical significance (i.e. the darker-shaded area).

The contour-enhanced funnel plot showed evidence of asymmetry (Fig. 5). However, the darker-shaded area, where “missing studies” would be expected in order to correct for visually detected asymmetry, lay within the significant regions of the plot. This would suggest that the asymmetry observed might not be due to publication bias based on statistical significance. This notion was confirmed by a non-significant Egger’s test (bias coefficient = −0.25, 95% CI: −3.43 to 2.92; *P* = 0.856). Furthermore, applying the trim-and-fill method to this meta-analysis by using the metatrim command resulted in no studies needing to be filled.

## Discussion

Based on the huge success of the MAGIC study, NAC has recently become another research hotspot (in addition to surgery and AC) for treatment of operable gastric cancer. Unfortunately, the trials comparing NAC plus surgery with surgery alone have failed to demonstrate any survival benefit in operable gastric cancer patients^19^,^20^. The related meta-analyses also failed to find a correlation, and there were some weaknesses in the inclusion criteria and statistical analyses in those studies^9^,^10^,^11^. In the present meta-analysis, we discovered convincingly that the treatment strategy of PC plus surgery in the treatment arm verified the statistical significance of the effect on improving OS in contrast to AC following surgery treatment in the control arm. In other words, the addition of NAC to AC (i.e. PC) made the death hazard in operable Asian gastric cancer patients fall dramatically by more than half (HR = 0.48, 95% CI: 0.35–0.67; *P* < 0.001) compared with AC alone. While the HR of OS for AC over surgery in gastric cancer was much higher (0.82, 95% CI: 0.76–0.90; *P* < 0.001), as pooled in another classical meta-analysis by Paoletti *et al.*^30^. However, the pooled HR of the two trials comparing NAC plus surgery with surgery alone did not show any OS advantage for NAC. The great diversity between these outcomes shows that NAC alone is not enough and AC alone is not good enough to definitely improve the OS in patients with gastric cancer, for NAC plus surgery is not superior to surgery alone, and AC is inferior to PC, as discovered in this meta-analysis.

It should be noted that the three trials suggesting the superiority of PC over AC were all carried out in east Asia where AC following D2 gastrectomy is a standard therapeutic strategy based on two pivotal RCTs (ACTS-GC^5^ and CLASSIC^7^), but NAC for resectable gastric cancer is not accepted as widely as it is in Europe. A large number of Asian doctors do not have a clear idea of the effects of NAC. They tend to be suspicious of its effect and side effect. Our present results provide hard evidence to help dispel the doubts about NAC and to promote the implementation of NAC in combination with AC to achieve better survival outcomes safely in gastric cancer patients, without significantly increasing the postoperative morbidity and mortality. Moreover, it is necessary to conduct large RCTs comparing PC with AC in gastric cancer to validate our conclusion. The results of such studies might change the current chemotherapeutic strategy and lead to further survival advantage for gastric cancer patients. Coincidentally, our proposal is consistent with the recent viewpoints of some Asian scholars^31^,^32^.

It is not unexpected that the other two trials conducted in Europe comparing NAC with surgery obtained negative results in our survival analysis. MAGIC^3^, FFCD9703^27^, and several recent studies^8^,^33^ have all produced results strongly suggesting that PC rather than surgery alone is beneficial for resectable gastric cancer. Thus, we propose that a combined strategy of NAC plus AC is as yet the best treatment for operable gastric cancer. Treatment strategy in both Asia and Europe appear to have reached consensus on this issue. However, it should be noted that in Europe results are only available for the comparison of PC with surgery, and not for the comparison of PC with AC^3^,^26^,^27^. Thus, large RCTs should also be conducted in Europe to address this question. In addition, there is no Asian trial focusing on the effectiveness on NAC plus surgery versus surgery alone yet. However, considering the wide acceptance of AC in Asia and the negative results of NAC alone in Europe, it might make little sense—and would hardly meet the ethical requirements to perform such research in Asia.

Regimens of all the trials included in our analysis contained fluorouracil (5-FU) or its derivatives, and some contained platinum, anthracycline or taxane. Based on the results of subgroup analysis on NAC regimens, we concluded that gastric cancer patients might benefit from NAC regimens containing platinum and fluorouracil. Unfortunately, the trials included in this meta-analysis did not contain the current generally-accepted regimens such as FOLFOX, XELOX, or S-1. A Japanese phase-II clinical trial suggested that a fluorouracil-free TC regimen (paclitaxel and cisplatin) for NAC might also be effective and safe in locally advanced gastric cancers^34^. As it stands, large RCTs are still needed to confirm the effectiveness and relevance of different regimens of NAC.

In terms of PFS, the only trial in this meta-analysis to compare NAC with surgery obtained a negative result^20^. The pooled HR for PFS in two other trials comparing PC with surgery was 0.66 (95% CI: 0.55–0.78; *P* < 0.001)^3^,^27^, which was much lower than that for pure AC versus surgery (HR = 0.82; 95% CI: 0.75–0.90; *P* < 0.001)^30^. When analyzing horizontally, we found that PC was superior to AC or NAC alone in improving PFS for resectable gastric cancer patients, which was consistent with the result for OS. The small number of studies included showed the need for large-scale RCTs for comparison of PC with AC in PFS.

The R0 resection rate is a crucial factor related to prognosis, and there was also controversy on the effect of NAC to increase it^9^,^11^,^35^. We found that NAC significantly improved the R0 resection rate, and subgroup analysis revealed that the R0 resection rate was significantly improved in Asian countries but not in non-Asian regions. This discrepancy might result from differences between regions in surgical procedures, in the extent of resection, or in pathological examinations. Tumor stage is another significant prognostic factor for gastric cancer patients. D’Ugo *et al.* reported that 3-year survival of gastric cancer patients after NAC was significantly related to tumor down-staging, and the patients with successful R0 resections saw an improvement in survival^36^. Because the baseline of the NAC group and the control group was balanced in our study, the pathological examination after surgery could reflect the down-staging effect of NAC. The percentages of both T0-2 and N0 staging were significantly higher in the NAC group indicating a down-staging effect of NAC for locally advanced gastric cancer. This was consistent with the work of Li *et al.* who showed that NAC significantly reduces tumor stage in pT0-2 (OR = 1.71, 95% CI: 1.26–2.33)^9^.

**Figure 6:**
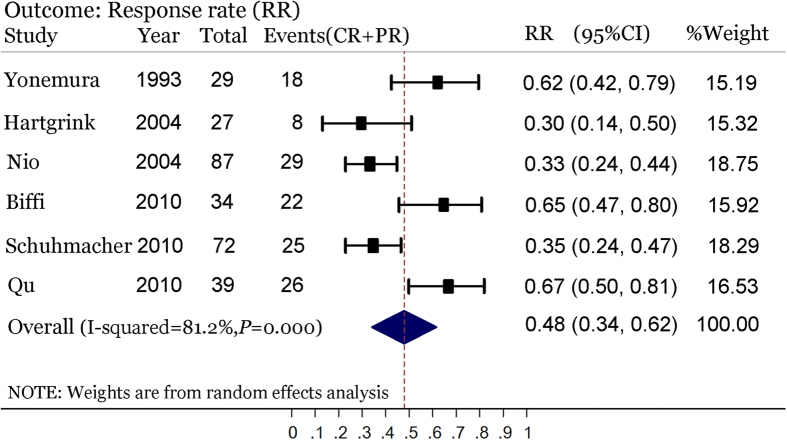
Pooled response rate of NAC for resectable gastric cancer in six NAC-containing RCTs using a random effect model.

On the whole, NAC alone could down-stage the primary tumor and improve the R0 resection rate but this might not translate into a long-term survival benefit. However, NAC followed by AC (i.e., PC) could significantly increase the survival advantage in resectable gastric cancer patients. So, why would this distinction exist? We combined the available response rates of NAC in six trials^19^,^20^,^22^,^24^,^25^,^28^ and found that the pooled response rate of NAC was 0.48 (95% CI: 0.34–0.62; Fig. 6), i.e., almost half of the patients showed no response to NAC. Blank *et al.* has reported a positive relationship between response rate to NAC and R0 resection rate, and even median OS^37^. In this meta-analysis, the R0 resection rates, down-staging of primary tumor or lymph node in the NAC arm were increased by 7.3%, 6.7%, and 10.2%, respectively. These improvements were significant but limited—possibly related to the limitation of the response to NAC. Thus, the possible limitation of NAC might be overcome by the following AC, in light of the positive effect of AC in improving the survival of gastric cancer patients. Furthermore, we have found that PC can significantly reduce the rate of distant metastasis in gastric cancer patients, while NAC alone can’t. This might be related to the effect of AC in preventing recurrence and metastasis although clinical trials have shown divergent results on this issue^38^,^39^,^40^,^41^,^42^,^43^. We speculate, therefore, that PC might be more effective in controlling recurrence and metastasis of operable gastric cancer than NAC alone, and this is in accordance with the outcomes of OS and PFS. However, this speculation should be verified by further study because of its limitation of scale and geographic region. In addition, a NAC regimen with poor effect could be substituted by another one during AC period^24^,^25^. The modifying effect could to some extent compensate for the limitations of NAC and improve the overall efficacy. The combination of NAC and AC could, therefore, lead to a synergistic effect, and this means that PC could maximize the survival benefit in operable gastric cancer patients.

Chemoradiotherapy is recommended as the standard treatment strategy for locally advanced gastric cancer in the US based on the US Intergroup-0116 trial^4^. In Asia, the ARTIST study suggested that postoperative radiotherapy might not be necessary in combination with AC after D2 gastrectomy, but it did significantly prolong DFS in patients with pathological lymph node-positive disease^44^. A Chinese study showed that adjuvant chemoradiotherapy (ACR) was associated with an increase in the median duration of recurrence-free survival compared to AC, and that this effect occurred regardless of whether or not there was lymph node metastasis^45^. In western countries, a prospective RCT (CRITICS)^46^ is being carried out with the intention of comparing NAC plus ACR with PC in gastric cancer patients. However, there is no large RCT focusing on comparison of PC with ACR. It is urgent that large RCTs comparing PC with radiotherapy-containing treatment be performed to find the optimal multimodal treatment strategy for operable gastric cancers.

This meta-analysis has the following limitations. First, the regimens used in the trials varied greatly. Second, the quantity and quality of RCTs comparing NAC with surgery were limited making it less practical to analyze the effect on OS of NAC alone. Third, the trial by Yonemura^22^ enrolled some stage-IV gastric cancer patients, and Nio^24^ used randomized consent design in his trial (i.e. grouping was partly decided by the patients). This resulted in a baseline that was not well balanced and which might account for underestimation of the effect of NAC.

In conclusion, our meta-analysis supports the use of PC in Asian countries as well as in Europe. We also suggest that large international RCTs comparing PC with AC should be conducted to further verify the effect of PC in operable gastric cancer patients. Current chemotherapy regimens are recommended in these trials and could be adjusted at the AC stage according to the response to NAC. It is also necessary to perform large RCTs comparing PC with radiotherapy-containing treatment to find the optimal multimodal treatment strategy for operable gastric cancers. The final results will help to guide and change clinical practice in gastric cancer treatment.

## Additional Information

**How to cite this article**: Yang, Y. *et al.* Perioperative chemotherapy more of a benefit for overall survival than adjuvant chemotherapy for operable gastric cancer: an updated Meta-analysis. *Sci. Rep.* **5**, 12850; doi: 10.1038/srep12850 (2015).

## Supplementary Material

Supplementary Information

## Figures and Tables

**Table 1:** Characteristics of all the trials included in the meta-analysis.

**Study**	**Country**	**No. of patients (NAC/ control arm)**	**Regimen**				**Surgical procedure**	**Tumor**		**Median follow-up (m)**	**OS**	**PFS**	**5-year survival**
			**NAC arm**		**Control arm**								
			**Pre-operative**	**Post-operative**	**Pre-operative**	**Post-operative**		**site**	**stage**		**Hazard ratio (95% CIs)**	**Hazard ratio (95% CIs)**	**(NAC /control)**
**Neoadjuvant chemotherapy plus surgery** ***vs.*** **Surgery**													
Masuyama M, 1994^16^	Japan	24/74	EAP-II^[1]^				—	—	—	—	—	—	—
Kobayashi O, 1994^17^	Japan	47/125	Aclarubicin^[2]^				D2	—	II/III	—	—	—	59.6%/48.0%
Wang X, 2000^18^	China	30/30	FPLC^[3]^				—	—	—	60	—	—	40.0%/23.3%
Hartgrink H, 2004^19^	Netherlands	29/30	FAMTX^[4]^				D1	stomach	II/III	83	1.51 (0.84, 2.74)**	—	20.7%/33.3%
Schuhmacher C, 2010^20^	Germany	72/72	FP^[5]^				D1 (7) or D2 (130)	stomach/EGJ*	II~IV	52.8	0.84 (0.52, 1.35)	0.76 (0.49, 1.16)	—
**Perioperative chemotherapy plus surgery*****vs.*** **surgery plus adjuvant chemotherapy**													
Kang Y, 1996^21^	South Korea	53/54	PEF^[6]^	PEF^[7]^		PEF	—	—	—	—	—	—	—
Yonemura Y, 1993^22^	Japan	29/26	PMUE^[8]^	PMUE^[8]^		PMUE	D2, D3	stomach	IV	24	0.49 (0.25, 0.94)**	—	—
Kobayashi T, 2000^23^	Japan	91/80	5′-DFUR^[9]^	5′-DFUR + MMC^[10]^		5′-DFUR + MMC	—	—	—	—	—	—	63.7%/65.0%
Nio Y, 2004^24^	Japan	102/193	UFT^[11]^	UFT or CF + UFT^[12]^		UFT or ECF + UFT	D1 (131) D2 (150)	—	I~IV	83	0.53 (0.33, 0.86)	—	—
Qu J, 2010^25^	China	39/39	PTX + FOLFOX4^[13]^	PTX + FOLFOX4 or ECF^[14]^		PTX + FOLFOX4 or ECF	—	stomach/EGJ	II/III	≥ 48	—	—	—
**Perioperative chemotherapy plus surgery** ***vs.*** **Surgery**													
Lygidakis N, 1999^26^	Greece	39/19	FAM^[15]^	FAM^[15]^			—	—	—	—	—	—	—
Cunningham D, 2006^3^	UK	250/253	ECF^[16]^	ECF^[16]^			D1 or D2	stomach/lower esophagus/EGJ	II/III	48	0.74 (0.59, 0.93)	0.66 (0.53, 0.81)	—
Ychou M, 2011^27^	France	113/111	FP^[17]^	FP^[18]^			D2	stomach/lower esophagus/EGJ	—	68.4	0.69 (0.50, 0.95)	0.65 (0.48, 0.89)	—
**Neoadjuvant chemotherapy plus surgery** ***vs.*** **surgery plus adjuvant chemotherapy**													
Biffi R, 2010^28^	Italy	34/35	TCF^[19]^			TCF^[20]^	D1, D2 or D3	stomach	IB/II/III	—	—	—	—

^*^EGJ refers to esophagogastric junction.

^**^Data were extracted from the Kaplan-Meier curves.

[1] Etoposide 100 mg, Epirubicin 20 mg, Carboplatin 100 mg; intra-arterial injection. [2] Medicinal lymph node dissection using aclarubicin adsorbed onto activated carbon. [3] Fluorouracil polyphase liposome composite. [4] Methotrexate 1,500 mg/m^2^, d2; 5-fluorouracil (5-FU) 1,500 mg/m^2^, d2; Leucovorin 30 mg, d3-d4; Doxorubicin 30 mg/m^2^, d15; every 4 weeks,  ≤ 4 cycles. [5] Cisplatin 50 mg/m^2^, d1, d15, d29; d-L-folinic acid 500 mg/m^2^, d1, d8, d15, d22, d29, d36; Fluorouracil 2,000 mg/m^2^, d1, d8, d15, d22, d29, d36; every 48 days, 2 cycles. [6] Cisplatin 20 mg/m^2^ iv d1–d5; VP16 100 mg/m^2^ iv d1, d3, d5; 5-FU 800 mg/m^2^ iv 12 h infusion d1–d5, repeated every 3 weeks. [7] The same regimen as no. 6; 3 cycles for curative resection and 6 cycles for non-curative resection. [8] Cisplatin 75 mg/m^2^, d1; Mitomycin-C 10 mg, d1; Etoposide 50 mg, d3–d5; every 3 weeks; UFT 400 mg daily. [9] 5′-DFUR 610 mg/m^2^/day, po ≥ 10 d. [10] MMC, iv, d1, d2; 5′-DFUR, po for 2 years. [11] UFT 6–8 mg/kg/day orally within 1 h after meals. [12] Stage I–III: UFT for 1–3 years; Stage IV: 1–4 courses of CDDP, 5-FU and Epirubicin (EPI) (FPEPIR regimen) and then oral UFT daily. [13] Paclitaxel 135 mg/m^2^, d1; Oxaliplatin 85 mg/m^2^, d1; Leucovorin 200 mg/m^2^, d1, d2; 5-FU 400 mg/m^2^, iv d1, d2; 600 mg/m^2^, civ 22 h, d1, d2; every 2 weeks, 3 cycles. [14] PTX + FOLFOX4: the same as no. 13. If disease progressed, ECF for 3 cycles. [15] Mitomycin-C, 5-FU, Leucovorin, and Farmorubicin, 10 days before surgery. [16] Epirubicin 50 mg/m^2^, d1; Cisplatin 60 mg/m^2^, d1; Fluorouracil 200 mg/m^2^, d1–d21. [17] 5-FU 800 mg/m^2^, d1–5; Cisplatin 100 mg/m^2^, d1; every 28 days, 2–3 cycles. [18] The same regimen as no. 17, 3–4 cycles. [19] Docetaxel 75 mg/m^2^, d1; Cisplatin 75 mg/m^2^, d1; 5-FU 300 mg/m^2^, d1–d14; every 3 weeks, 2–4 cycles. [20] The same regimen as [19], 4 cycles.

**Table 2:** Quality assessment of the studies included.

**Study**	**Randomization**	**Allocation concealment**	**Blinding**	**Withdrawal and dropout**	**Selective reporting**	**Baseline**	**Quality level**
Cunningham D^3^	By data center	By phone call	No details	Well reported	Not mentioned	Identical baseline	A
Masuyama M^16^	No details	No details	No details	Not mentioned	Not mentioned	Not mentioned	B
Kobayashi O^17^	No details	No details	No details	Not mentioned	Not mentioned	Not mentioned	B
Wang X^18^	No details	No details	No details	Well reported	Not mentioned	Not mentioned	B
Hartgrink H^19^	By data center	By phone call	No	Well reported	Not mentioned	Identical baseline	A
Schuhmacher C^20^	No details	No details	No details	Well reported	Not mentioned	Different performance status	B
Kang Y^21^	No details	No details	No details	Not mentioned	Not mentioned	Not mentioned	B
Yonemura Y^22^	Well reported	Sealed envelope	No	Well reported	Not mentioned	Identical baseline	A
Kobayashi T^23^	No details	No details	No details	Not mentioned	Not mentioned	Not mentioned	B
Nio Y^24^	Single-consent	No	No	Well reported	Not mentioned	Different stage and histological grade	C
Qu J^25^	Random digits table	No details	No details	Well reported	Not mentioned	Identical baseline	A
Lygidakis N^26^	No details	No details	No details	Not mentioned	Not mentioned	Not mentioned	B
Ychou M^27^	Central randomization	By phone call	No details	Well reported	Not mentioned	Identical baseline	A
Biffi R^28^	No details	No details	No details	Well reported	Not mentioned	Identical baseline	B
