# Supplementary material for: Perioperative chemotherapy more of a benefit for overall survival than adjuvant chemotherapy for operable gastric cancer: an updated Meta-analysis
Source: Sci Rep. 2015 Aug 5;5:12850. doi: 10.1038/srep12850 (PMC4525358; doi:10.1038/srep12850)
Supplement: Supplementary Information [file srep12850-s1.pdf]

Perioperative chemotherapy more of a benefit for overall survival than adjuvant chemotherapy for operable gastric cancer: an updated Meta-analysis

Ya'nan Yang[1*], Xue Yin[1*], Lei Sheng[2*], Shan Xu[1], Lingling Dong[3] and Lian Liu[1]

[1] Department of Chemotherapy, Cancer Center, Qilu Hospital, Shandong University, Jinan, China

[2] Cancer Therapeutics Laboratory, Centre for Personalized Cancer Medicine, School of Medicine, University of Adelaide, Australia

[3] Department of Cancer, Weifang Traditional Chinese Medical Hospital, Weifang, China

Correspondence to: Lian Liu, Department of Chemotherapy, Cancer Center, Qilu Hospital, Shandong University, Jinan 250012, Shandong, P R China.

lianliu@sdu.edu.cn

*Y.Y., X.Y., and L.S. contributed equally to this work.

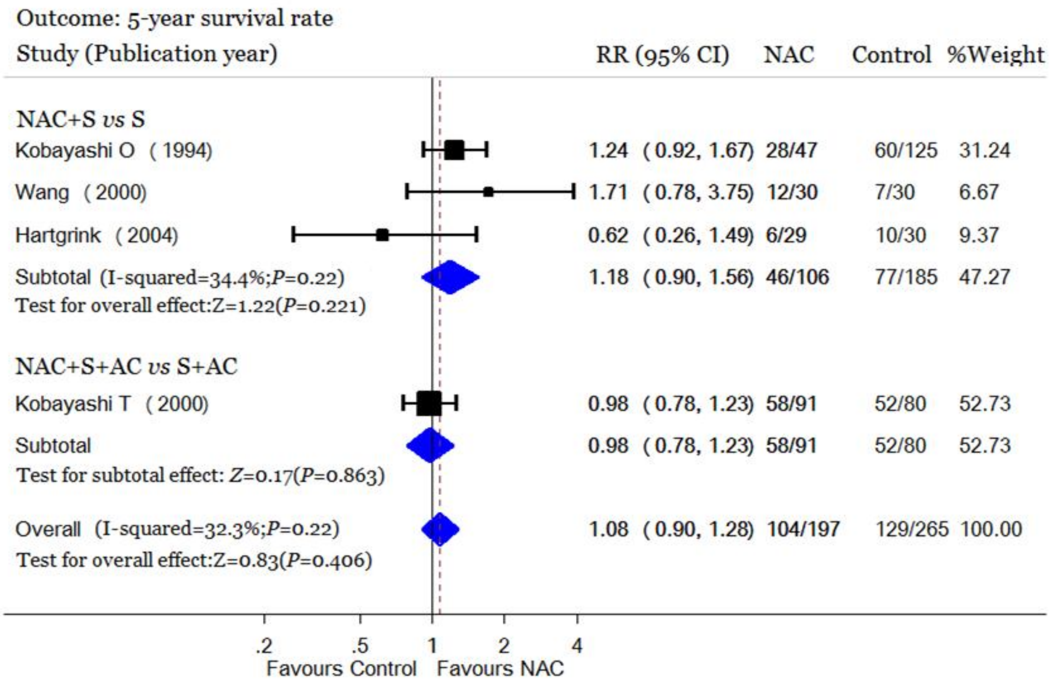

Supple figure 1. Forest plot showing 5-year survival rates of resectable gastric cancer patients in four NAC-containing RCTs.

The relative risk (RR) with 95% confidence interval (CI) for the effect of treatment on the 5-year survival rate is shown on a logarithmic scale using a random effect model.

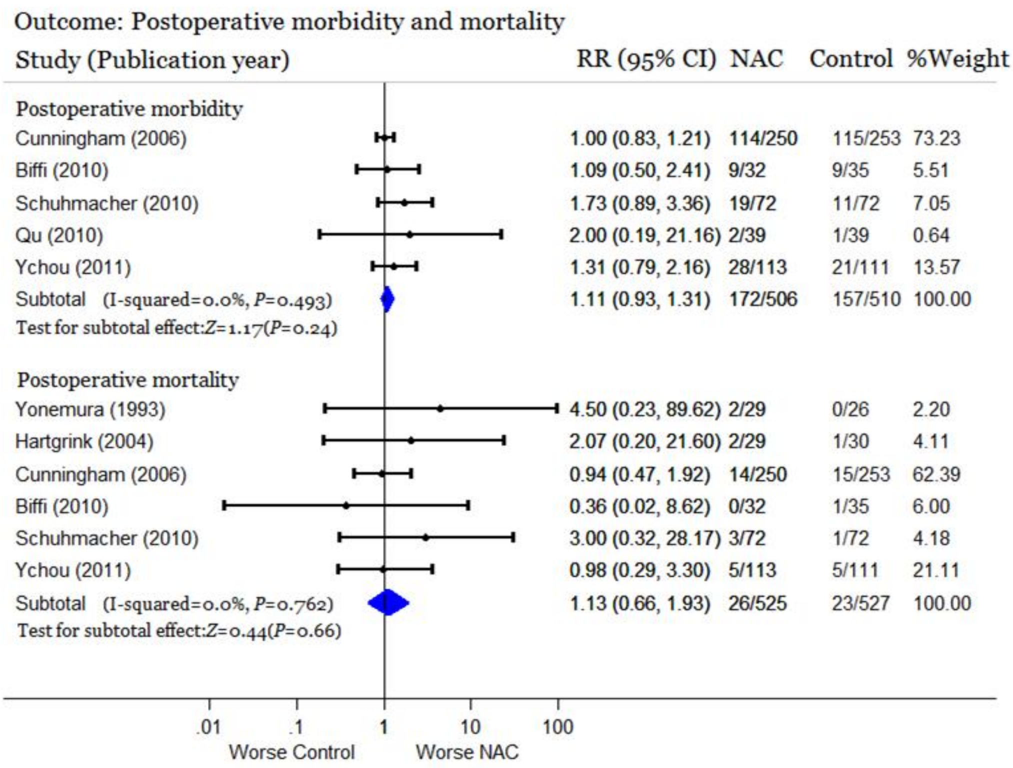

Supple figure 2. Forest plot showing postoperative morbidity and mortality of gastric cancer patients in the NAC-containing RCTs.

The relative risk (RR) with 95% confidence interval (CI) for effect of treatment on postoperative morbidity and mortality is shown on a logarithmic scale using a fixed effect model.
